# Divergence across the mitogenomes of *Branchinella kugenumaensis* (Anostraca: Thamnocephalidae) with implications for species delimitation

**DOI:** 10.1080/23802359.2021.1876537

**Published:** 2021-02-17

**Authors:** Xiaoyan Sun

**Affiliations:** State Key Laboratory of Palaeobiology and Stratigraphy, Nanjing Institute of Geology and Palaeontology and Center for Excellence in Life and Palaeoenvironment, Chinese Academy of Sciences, Nanjing, China

**Keywords:** Cryptic species, mitogenome, nonsynonymous codon substitutions, phylogenetics, hairpin structures

## Abstract

The complete mitochondrial genome of *Branchinella kugenumaensis* from Japan is sequenced, annotated, and compared with that of *B. kugenumaensis* from China. The mitogenome consists of 14,123 bp with a GC content of 31.83%. Features of both mitogenomes including the presence of regulatory elements in the control region, secondary structure features of tRNAs, and substitution patterns are described and discussed in an evolutionary framework. Comparative studies and genetic analyses indicate high levels of diversity between these two geographically separated populations of *B. kugenumaensis*, suggesting that they are probably separate species.

Genus *Branchinella* Sayce 1903 (Anostraca: Thamnocephalidae) is an important member of the macrofaunal community in aquatic environments. These freshwater crustaceans are exceptionally diverse and widespread in Europe, Asia, North and South America, southern Africa, and Australia (Belk and Brtek 1995; Brendonck and Belk 1997; Remigio et al. 2003; Rogers et al. 2013). Large scale phylogenies of the Australian *Branchinella* were reconstructed using a segment of the mitochondrial ribosomal RNA (16S) gene, and the presence of at least three new cryptic species was recovered (Remigio et al. 2003; Pinceel et al. 2013). Yang and Chen (2020) first published the complete mitogenome of *B. kugenumaensis* Ishikawa, 1895 from Zhejiang Province, China. Despite the high species diversity, however, mitogenomes of *Branchinella* remain scarce. In this article, the complete mitogenome of *B. kugenumaensis* sampled from Japan was determined. A comparative analysis in an evolutionary framework was performed for investigating the evidence of cryptic species at the complete genome level.

Specimens of *B. kugenumaensis* were collected from a paddy field (134°26′ E, 34°03′ N) near Naruto City, Tokushima Prefecture, Japan. The Voucher specimens (No. BLKNJA01) were deposited in the State Key Laboratory of Palaeobiology and Stratigraphy, Nanjing Institute of Geology and Paleontology, Chinese Academy of Sciences, Nanjing, China. Total DNA was extracted from one adult using the DNeasy tissue kit (Qiagen, Hilden, Germany). The complete mitogenome sequence was determined by long-polymerase chain reaction with conserved primers and sequenced with primer walking using flanking sequences. Overlapped fragments obtained by sequencing were assembled into contigs and aligned using BioEdit 7.0.9.0 (Hall 1999). Annotation of mitogenome used MITOS v1 (Bernt et al. 2013). Thirteen protein-coding genes were extracted from each mitogenome and aligned using MEGA 7 (Kumar et al. 2016). The substitution patterns were obtained using DAMBE 6 version (Xia 2017). Bayesian inference (BI) was conducted MrBayes version 3.2 (Ronquist et al. 2012). Mean maximum likelihood distances were calculated using MEGA 7 (Kumar et al. 2016).

The total length of mitogenome of *B. kugenumaensis* from Japan (GenBank accession number: MW136376) is 14,123 bp (31.83% GC content), which is 15,127 bp in the Chinese population. This difference is mainly due to the total fraction of noncoding sequences, being 147 and 1182 bp, respectively. Detailed analysis of both genomes shows the presence of peculiar patterns in the control region of *B. kugenumaensis* from China, e.g. hairpin structures, poly-T tract, AT-rich, and conserved motifs. A total of 94 substitutions occur between the two tRNA sets. The majority of substitutions (48 sites) are observed in the stems. Six PCGs show different lengths between the two genomes: *atp8* and *nad1* differ by 15 bp, *nad5* by 13 bp, *atp6* by 11 bp, *nad2* by 8 bp, and *nad4L* by 6 bp. Percent sequence divergence between the two genomes calculated for each aligned PCG ranges from 16.36% (*cox1*) to 32.05% (*atp8*) and total number of nonsynonymous codon substitutions ranges from 5 (*cox2*) to 87(*nad5*), which constitutes a high level of diversity between the two *B. kugenumaensis* populations. The unusually high variation observed in the mitogenomes of two geographically separated populations prompted the question whether or not Asian *B. kugenumaensis* were separate species. Considering the scarce number of mitogenomes, the 16S rRNA gene was utilized to construct the phylogeny of genus *Branchinella*, and to calculate the genetic distances. The inferred phylogenetic tree based on the 16S rRNA sequences places *B. kugenumaensis* of Japan within the Asian *Branchinella* clade, as the sister to *B. kugenumaensis* of China. The phylogenetic relationships within Australian species are consistent with the topologies of Remigio et al. (2003) and Pinceel et al. (2013). ML-corrected genetic distances within 16 16S rRNA sequences of *Branchinella* range from 2.9 to 22.8%. The overall average divergence among the 16S rRNA of the 16 *Branchinella* species in this work is 11.9 ± 2.1%, while the two populations of *B. kugenumaensis* exhibit high genetic distance: 12.8 ± 2.3%. Similar levels of divergence are also found in comparisons performed on the *cox1* bar-coding fragment. To sum up, comparative analyses recover high levels of diversity between two geographically separated populations of *B. kugenumaensis*. Therefore, it is reasonable to suggest that these two populations of *B. kugenumaensis* probably belong to two different cryptic species, but this hypothesis needs to be thoroughly tested by more sampling from Asia coupled with more molecular data assessment and morphological characteristics to define appropriate conservation units.

## Data Availability

All relevant data can be obtained free of charge on NCBI through GeneBank No. MW136376 (NCBI https://www.ncbi.nlm.nih.gov/nuccore/MW136376). Bayesian inference tree depicting the phylogenetic (numbers at the nodes are Bayesian posterior probabilities). Geographical distributions of Branchinella are represented as Australian: green, Asian: yellow, Indian: purple, Japanese: blue, and Chinese: red.
